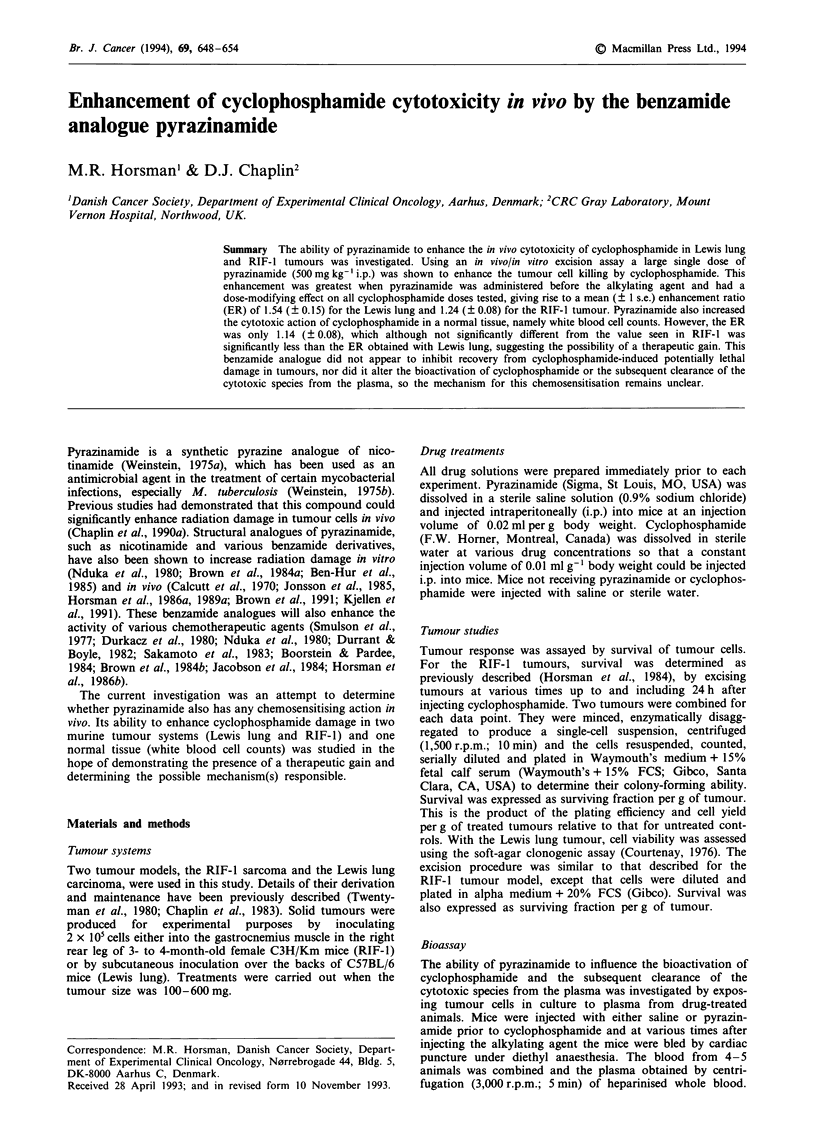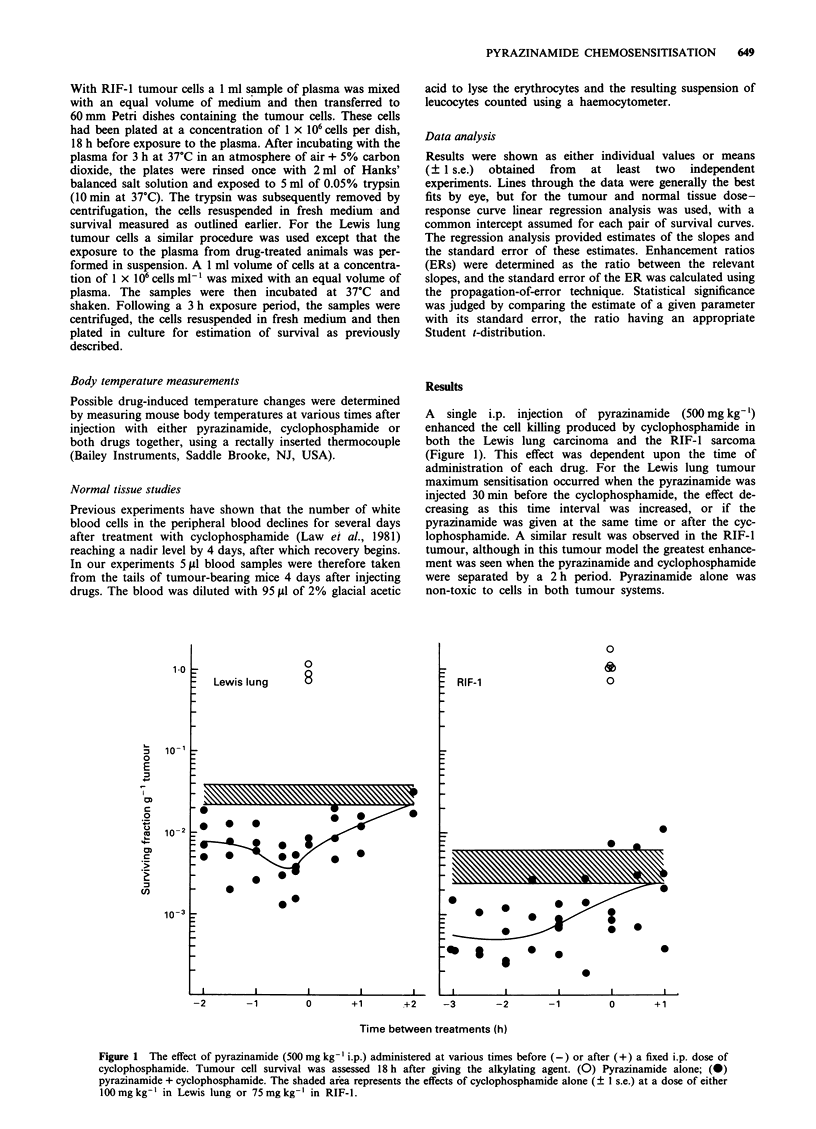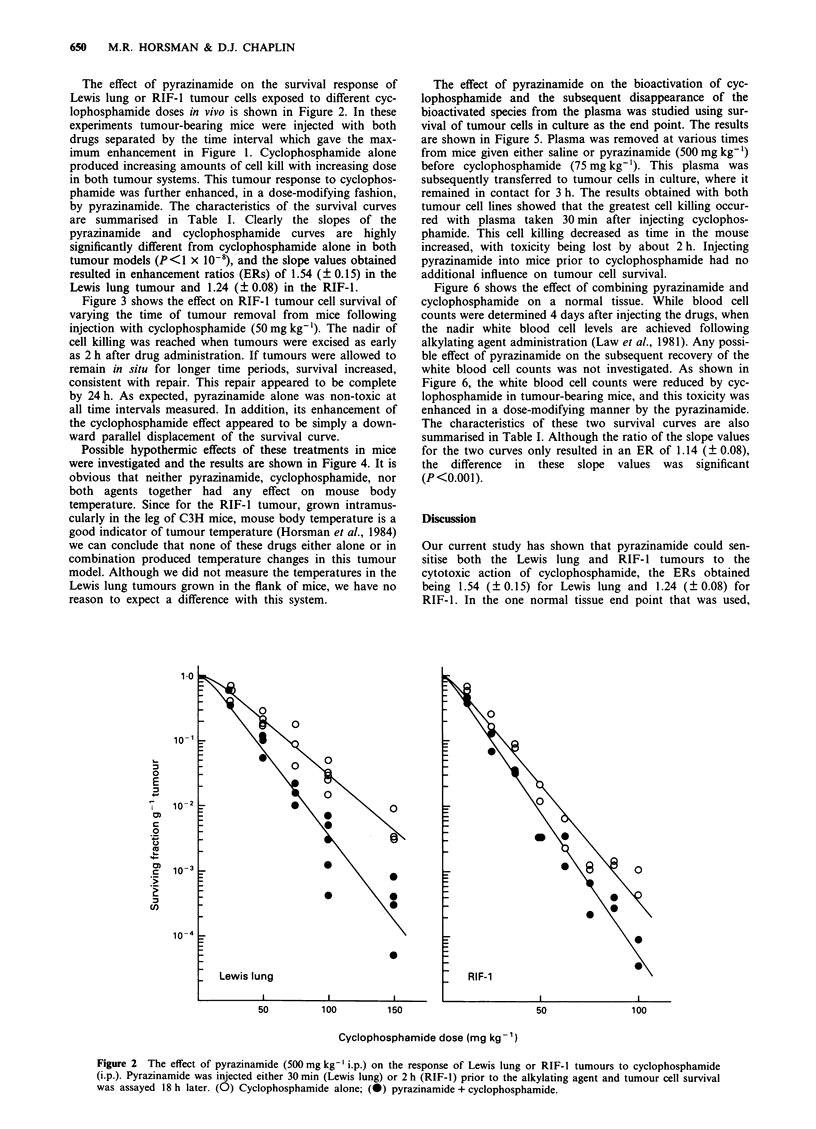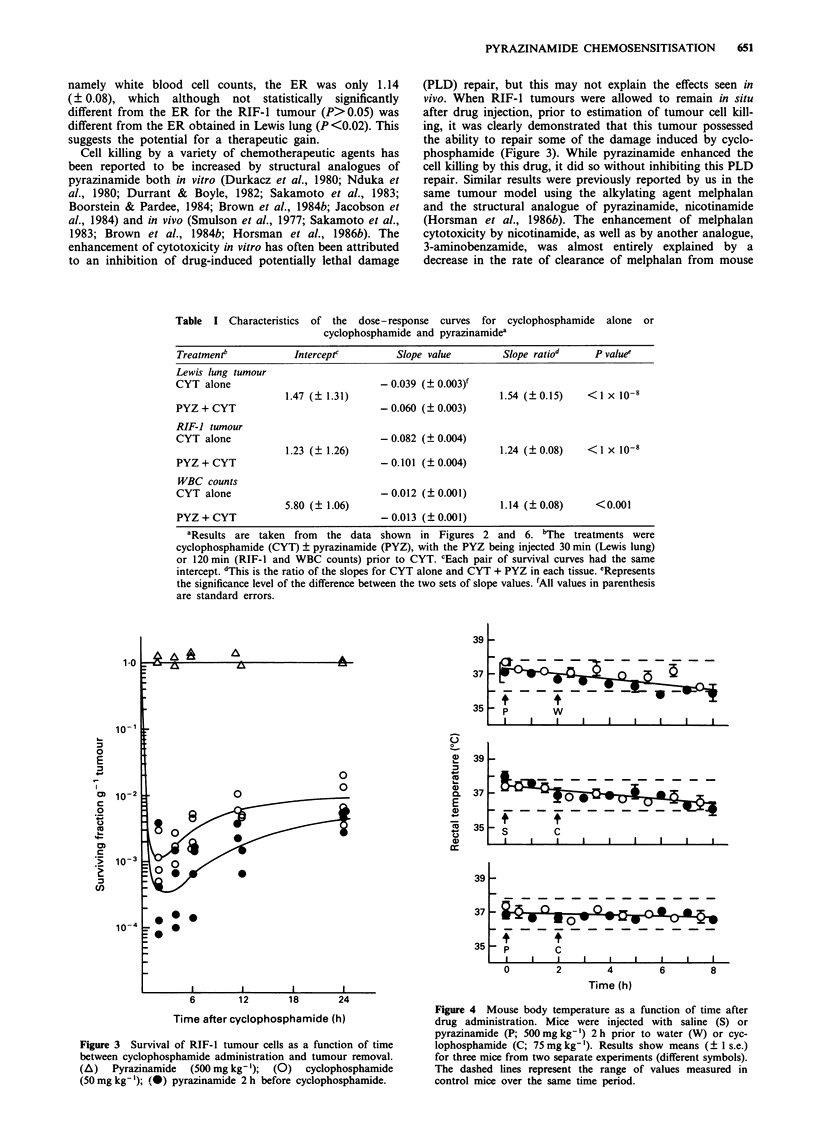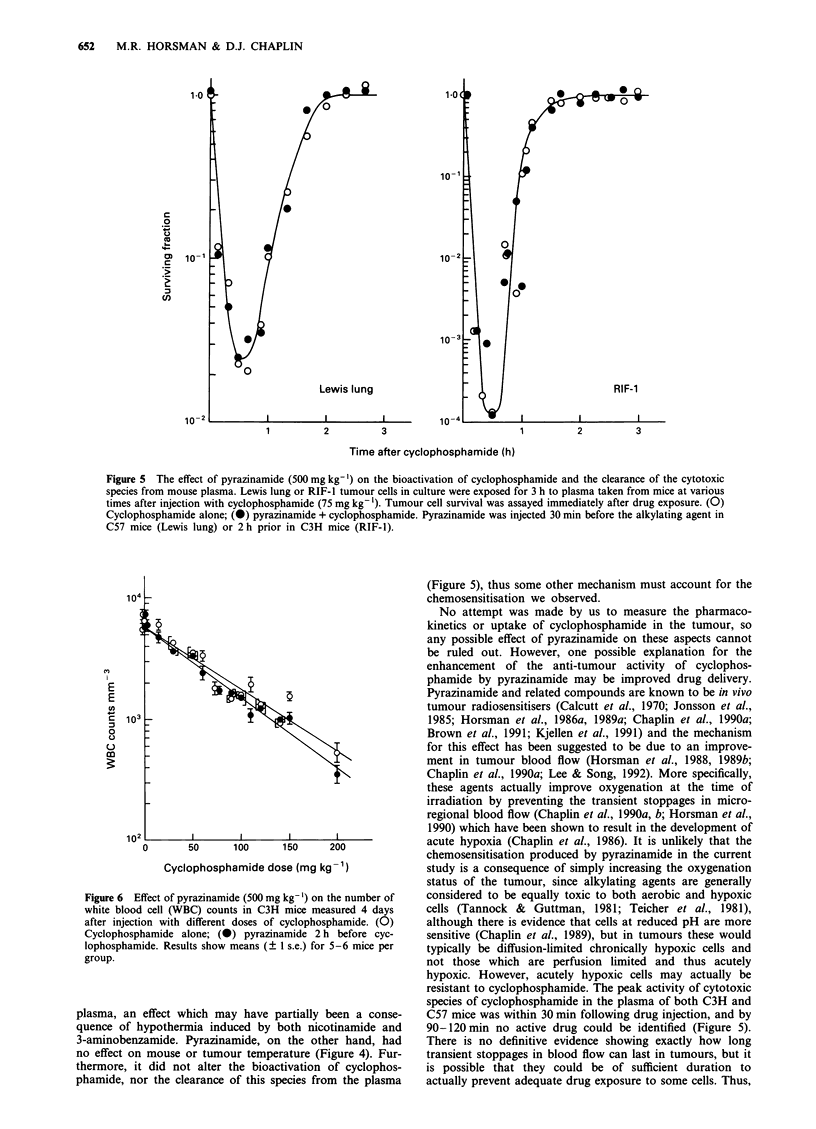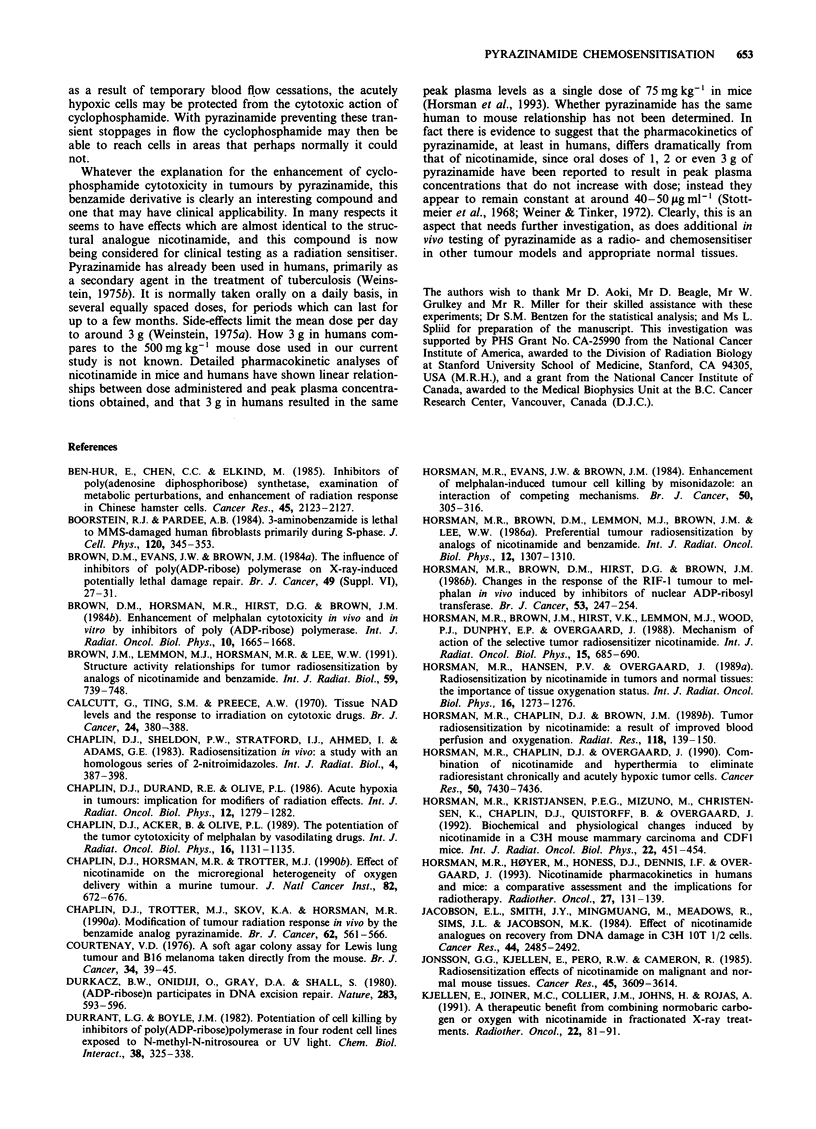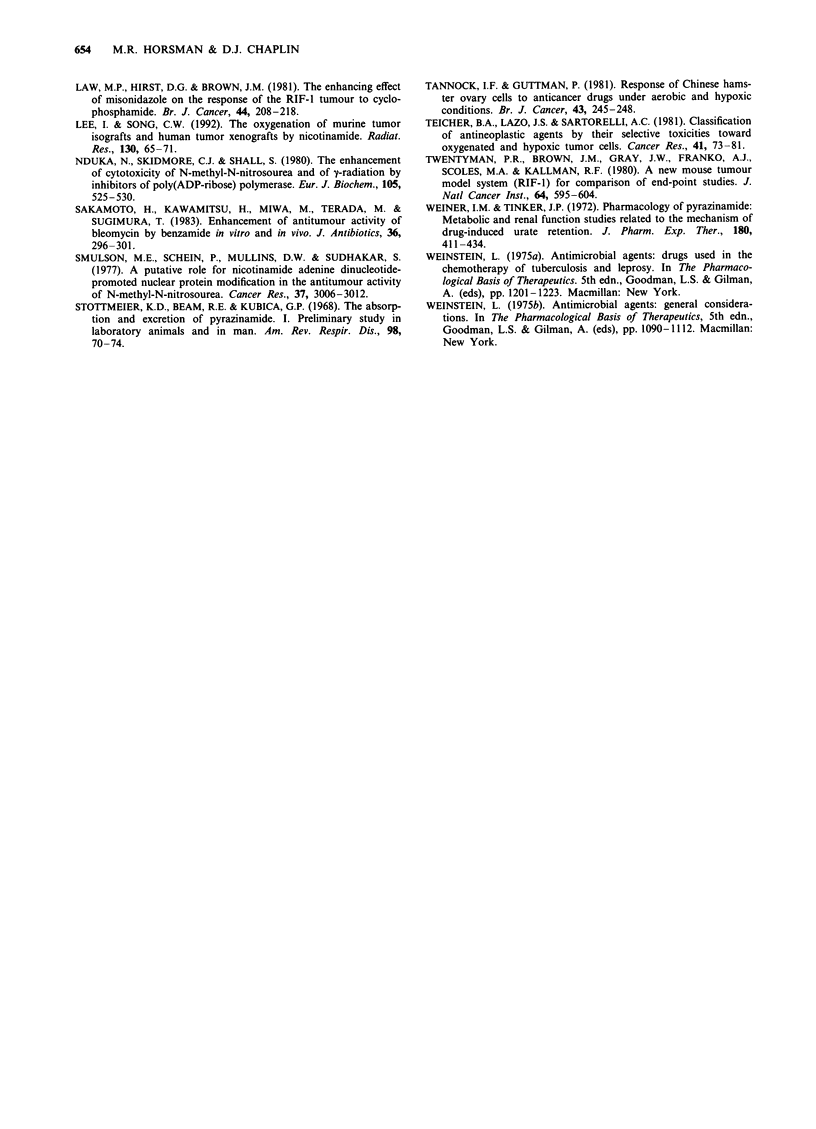# Enhancement of cyclophosphamide cytotoxicity in vivo by the benzamide analogue pyrazinamide.

**DOI:** 10.1038/bjc.1994.126

**Published:** 1994-04

**Authors:** M. R. Horsman, D. J. Chaplin

**Affiliations:** Danish Cancer Society, Department of Experimental Clinical Oncology, Aarhus.

## Abstract

The ability of pyrazinamide to enhance the in vivo cytotoxicity of cyclophosphamide in Lewis lung and RIF-1 tumours was investigated. Using an in vivo/in vitro excision assay a large single dose of pyrazinamide (500 mg kg-1 i.p.) was shown to enhance the tumour cell killing by cyclophosphamide. This enhancement was greatest when pyrazinamide was administered before the alkylating agent and had a dose-modifying effect on all cyclophosphamide doses tested, giving rise to a mean (+/- 1 s.e.) enhancement ratio (ER) of 1.54 (+/- 0.15) for the Lewis lung and 1.24 (+/- 0.08) for the RIF-1 tumour. Pyrazinamide also increased the cytotoxic action of cyclophosphamide in a normal tissue, namely white blood cell counts. However, the ER was only 1.14 (+/- 0.08), which although not significantly different from the value seen in RIF-1 was significantly less than the ER obtained with Lewis lung, suggesting the possibility of a therapeutic gain. This benzamide analogue did not appear to inhibit recovery from cyclophosphamide-induced potentially lethal damage in tumours, nor did it alter the bioactivation of cyclophosphamide or the subsequent clearance of the cytotoxic species from the plasma, so the mechanism for this chemosensitisation remains unclear.


Br. J. Cancer (1994), 69, 648-654 © Macmillan Press Ltd., 1994

### Enhancement of cyclophosphamide cytotoxicity *in vivo* by the benzamide analogue pyrazinamide

M.R. Horsman[1] & D.J. Chaplin[2]

[1]*Danish Cancer Society, Department of Experimental Clinical Oncology, Aarhus, Denmark;* [2]*CRC Gray Laboratory, Mount Vernon Hospital, Northwood, UK.*

**Summary** The ability of pyrazinamide to enhance the *in vivo* cytotoxicity of cyclophosphamide in Lewis lung and RIF-1 tumours was investigated. Using an *in vivo/in vitro* excision assay a large single dose of pyrazinamide (500 mg $kg^{-1}$ i.p.) was shown to enhance the tumour cell killing by cyclophosphamide. This enhancement was greatest when pyrazinamide was administered before the alkylating agent and had a dose-modifying effect on all cyclophosphamide doses tested, giving rise to a mean (± 1 s.e.) enhancement ratio (ER) of 1.54 (± 0.15) for the Lewis lung and 1.24 (± 0.08) for the RIF-1 tumour. Pyrazinamide also increased the cytotoxic action of cyclophosphamide in a normal tissue, namely white blood cell counts. However, the ER was only 1.14 (± 0.08), which although not significantly different from the value seen in RIF-1 was significantly less than the ER obtained with Lewis lung, suggesting the possibility of a therapeutic gain. This benzamide analogue did not appear to inhibit recovery from cyclophosphamide-induced potentially lethal damage in tumours, nor did it alter the bioactivation of cyclophosphamide or the subsequent clearance of the cytotoxic species from the plasma, so the mechanism for this chemosensitisation remains unclear.

Pyrazinamide is a synthetic pyrazine analogue of nicotinamide (Weinstein, 1975*a*), which has been used as an antimicrobial agent in the treatment of certain mycobacterial infections, especially *M. tuberculosis* (Weinstein, 1975*b*). Previous studies had demonstrated that this compound could significantly enhance radiation damage in tumour cells *in vivo* (Chaplin *et al.*, 1990*a*). Structural analogues of pyrazinamide, such as nicotinamide and various benzamide derivatives, have also been shown to increase radiation damage *in vitro* (Nduka *et al.*, 1980; Brown *et al.*, 1984*a*; Ben-Hur *et al.*, 1985) and *in vivo* (Calcutt *et al.*, 1970; Jonsson *et al.*, 1985, Horsman *et al.*, 1986*a*, 1989*a*; Brown *et al.*, 1991; Kjellen *et al.*, 1991). These benzamide analogues will also enhance the activity of various chemotherapeutic agents (Smulson *et al.*, 1977; Durkacz *et al.*, 1980; Nduka *et al.*, 1980; Durrant & Boyle, 1982; Sakamoto *et al.*, 1983; Boorstein & Pardee, 1984; Brown *et al.*, 1984*b*; Jacobson *et al.*, 1984; Horsman *et al.*, 1986*b*).

The current investigation was an attempt to determine whether pyrazinamide also has any chemosensitising action *in vivo*. Its ability to enhance cyclophosphamide damage in two murine tumour systems (Lewis lung and RIF-1) and one normal tissue (white blood cell counts) was studied in the hope of demonstrating the presence of a therapeutic gain and determining the possible mechanism(s) responsible.

### Materials and methods

### *Tumour systems*

Two tumour models, the RIF-1 sarcoma and the Lewis lung carcinoma, were used in this study. Details of their derivation and maintenance have been previously described (Twentyman *et al.*, 1980; Chaplin *et al.*, 1983). Solid tumours were produced for experimental purposes by inoculating $2 \times 10^5$ cells either into the gastrocnemius muscle in the right rear leg of 3- to 4-month-old female C3H/Km mice (RIF-1) or by subcutaneous inoculation over the backs of C57BL/6 mice (Lewis lung). Treatments were carried out when the tumour size was 100-600 mg.

Correspondence: M.R. Horsman, Danish Cancer Society, Department of Experimental Clinical Oncology, Nørrebrogade 44, Bldg. 5, DK-8000 Aarhus C, Denmark.
Received 28 April 1993; and in revised form 10 November 1993.

### *Drug treatments*

All drug solutions were prepared immediately prior to each experiment. Pyrazinamide (Sigma, St Louis, MO, USA) was dissolved in a sterile saline solution (0.9% sodium chloride) and injected intraperitoneally (i.p.) into mice at an injection volume of 0.02 ml per g body weight. Cyclophosphamide (F.W. Horner, Montreal, Canada) was dissolved in sterile water at various drug concentrations so that a constant injection volume of 0.01 ml $g^{-1}$ body weight could be injected i.p. into mice. Mice not receiving pyrazinamide or cyclophosphamide were injected with saline or sterile water.

### *Tumour studies*

Tumour response was assayed by survival of tumour cells. For the RIF-1 tumours, survival was determined as previously described (Horsman *et al.*, 1984), by excising tumours at various times up to and including 24 h after injecting cyclophosphamide. Two tumours were combined for each data point. They were minced, enzymatically disaggregated to produce a single-cell suspension, centrifuged (1,500 r.p.m.; 10 min) and the cells resuspended, counted, serially diluted and plated in Waymouth's medium + 15% fetal calf serum (Waymouth's + 15% FCS; Gibco, Santa Clara, CA, USA) to determine their colony-forming ability. Survival was expressed as surviving fraction per g of tumour. This is the product of the plating efficiency and cell yield per g of treated tumours relative to that for untreated controls. With the Lewis lung tumour, cell viability was assessed using the soft-agar clonogenic assay (Courtenay, 1976). The excision procedure was similar to that described for the RIF-1 tumour model, except that cells were diluted and plated in alpha medium + 20% FCS (Gibco). Survival was also expressed as surviving fraction per g of tumour.

### *Bioassay*

The ability of pyrazinamide to influence the bioactivation of cyclophosphamide and the subsequent clearance of the cytotoxic species from the plasma was investigated by exposing tumour cells in culture to plasma from drug-treated animals. Mice were injected with either saline or pyrazinamide prior to cyclophosphamide and at various times after injecting the alkylating agent the mice were bled by cardiac puncture under diethyl anaesthesia. The blood from 4-5 animals was combined and the plasma obtained by centrifugation (3,000 r.p.m.; 5 min) of heparinised whole blood.

With RIF-1 tumour cells a 1 ml sample of plasma was mixed with an equal volume of medium and then transferred to 60 mm Petri dishes containing the tumour cells. These cells had been plated at a concentration of $1 \times 10^6$ cells per dish, 18 h before exposure to the plasma. After incubating with the plasma for 3 h at 37°C in an atmosphere of air + 5% carbon dioxide, the plates were rinsed once with 2 ml of Hanks' balanced salt solution and exposed to 5 ml of 0.05% trypsin (10 min at 37°C). The trypsin was subsequently removed by centrifugation, the cells resuspended in fresh medium and survival measured as outlined earlier. For the Lewis lung tumour cells a similar procedure was used except that the exposure to the plasma from drug-treated animals was performed in suspension. A 1 ml volume of cells at a concentration of $1 \times 10^6$ cells $ml^{-1}$ was mixed with an equal volume of plasma. The samples were then incubated at 37°C and shaken. Following a 3 h exposure period, the samples were centrifuged, the cells resuspended in fresh medium and then plated in culture for estimation of survival as previously described.

### *Body temperature measurements*

Possible drug-induced temperature changes were determined by measuring mouse body temperatures at various times after injection with either pyrazinamide, cyclophosphamide or both drugs together, using a rectally inserted thermocouple (Bailey Instruments, Saddle Brooke, NJ, USA).

### *Normal tissue studies*

Previous experiments have shown that the number of white blood cells in the peripheral blood declines for several days after treatment with cyclophosphamide (Law *et al.*, 1981) reaching a nadir level by 4 days, after which recovery begins. In our experiments 5 µl blood samples were therefore taken from the tails of tumour-bearing mice 4 days after injecting drugs. The blood was diluted with 95 µl of 2% glacial acetic acid to lyse the erythrocytes and the resulting suspension of leucocytes counted using a haemocytometer.

### *Data analysis*

Results were shown as either individual values or means (± 1 s.e.) obtained from at least two independent experiments. Lines through the data were generally the best fits by eye, but for the tumour and normal tissue dose–response curve linear regression analysis was used, with a common intercept assumed for each pair of survival curves. The regression analysis provided estimates of the slopes and the standard error of these estimates. Enhancement ratios (ERs) were determined as the ratio between the relevant slopes, and the standard error of the ER was calculated using the propagation-of-error technique. Statistical significance was judged by comparing the estimate of a given parameter with its standard error, the ratio having an appropriate Student *t*-distribution.

### Results

A single i.p. injection of pyrazinamide (500 mg $kg^{-1}$) enhanced the cell killing produced by cyclophosphamide in both the Lewis lung carcinoma and the RIF-1 sarcoma (Figure 1). This effect was dependent upon the time of administration of each drug. For the Lewis lung tumour maximum sensitisation occurred when the pyrazinamide was injected 30 min before the cyclophosphamide, the effect decreasing as this time interval was increased, or if the pyrazinamide was given at the same time or after the cyclophosphamide. A similar result was observed in the RIF-1 tumour, although in this tumour model the greatest enhancement was seen when the pyrazinamide and cyclophosphamide were separated by a 2 h period. Pyrazinamide alone was non-toxic to cells in both tumour systems.

**Figure 1** The effect of pyrazinamide (500 mg $kg^{-1}$ i.p.) administered at various times before (−) or after (+) a fixed i.p. dose of cyclophosphamide. Tumour cell survival was assessed 18 h after giving the alkylating agent. (○) Pyrazinamide alone; (●) pyrazinamide + cyclophosphamide. The shaded area represents the effects of cyclophosphamide alone (± 1 s.e.) at a dose of either 100 mg $kg^{-1}$ in Lewis lung or 75 mg $kg^{-1}$ in RIF-1.

The effect of pyrazinamide on the survival response of Lewis lung or RIF-1 tumour cells exposed to different cyclophosphamide doses *in vivo* is shown in Figure 2. In these experiments tumour-bearing mice were injected with both drugs separated by the time interval which gave the maximum enhancement in Figure 1. Cyclophosphamide alone produced increasing amounts of cell kill with increasing dose in both tumour systems. This tumour response to cyclophosphamide was further enhanced, in a dose-modifying fashion, by pyrazinamide. The characteristics of the survival curves are summarised in Table I. Clearly the slopes of the pyrazinamide and cyclophosphamide curves are highly significantly different from cyclophosphamide alone in both tumour models ($P < 1 \times 10^{-8}$), and the slope values obtained resulted in enhancement ratios (ERs) of 1.54 ($\pm$ 0.15) in the Lewis lung tumour and 1.24 ($\pm$ 0.08) in the RIF-1.

Figure 3 shows the effect on RIF-1 tumour cell survival of varying the time of tumour removal from mice following injection with cyclophosphamide (50 mg $kg^{-1}$). The nadir of cell killing was reached when tumours were excised as early as 2 h after drug administration. If tumours were allowed to remain *in situ* for longer time periods, survival increased, consistent with repair. This repair appeared to be complete by 24 h. As expected, pyrazinamide alone was non-toxic at all time intervals measured. In addition, its enhancement of the cyclophosphamide effect appeared to be simply a downward parallel displacement of the survival curve.

Possible hypothermic effects of these treatments in mice were investigated and the results are shown in Figure 4. It is obvious that neither pyrazinamide, cyclophosphamide, nor both agents together had any effect on mouse body temperature. Since for the RIF-1 tumour, grown intramuscularly in the leg of C3H mice, mouse body temperature is a good indicator of tumour temperature (Horsman *et al.*, 1984) we can conclude that none of these drugs either alone or in combination produced temperature changes in this tumour model. Although we did not measure the temperatures in the Lewis lung tumours grown in the flank of mice, we have no reason to expect a difference with this system.

The effect of pyrazinamide on the bioactivation of cyclophosphamide and the subsequent disappearance of the bioactivated species from the plasma was studied using survival of tumour cells in culture as the end point. The results are shown in Figure 5. Plasma was removed at various times from mice given either saline or pyrazinamide (500 mg $kg^{-1}$) before cyclophosphamide (75 mg $kg^{-1}$). This plasma was subsequently transferred to tumour cells in culture, where it remained in contact for 3 h. The results obtained with both tumour cell lines showed that the greatest cell killing occurred with plasma taken 30 min after injecting cyclophosphamide. This cell killing decreased as time in the mouse increased, with toxicity being lost by about 2 h. Injecting pyrazinamide into mice prior to cyclophosphamide had no additional influence on tumour cell survival.

Figure 6 shows the effect of combining pyrazinamide and cyclophosphamide on a normal tissue. While blood cell counts were determined 4 days after injecting the drugs, when the nadir white blood cell levels are achieved following alkylating agent administration (Law *et al.*, 1981). Any possible effect of pyrazinamide on the subsequent recovery of the white blood cell counts was not investigated. As shown in Figure 6, the white blood cell counts were reduced by cyclophosphamide in tumour-bearing mice, and this toxicity was enhanced in a dose-modifying manner by the pyrazinamide. The characteristics of these two survival curves are also summarised in Table I. Although the ratio of the slope values for the two curves only resulted in an ER of 1.14 ($\pm$ 0.08), the difference in these slope values was significant ($P < 0.001$).

### Discussion

Our current study has shown that pyrazinamide could sensitise both the Lewis lung and RIF-1 tumours to the cytotoxic action of cyclophosphamide, the ERs obtained being 1.54 ($\pm$ 0.15) for Lewis lung and 1.24 ($\pm$ 0.08) for RIF-1. In the one normal tissue end point that was used,

**Figure 2** The effect of pyrazinamide (500 mg $kg^{-1}$ i.p.) on the response of Lewis lung or RIF-1 tumours to cyclophosphamide (i.p.). Pyrazinamide was injected either 30 min (Lewis lung) or 2 h (RIF-1) prior to the alkylating agent and tumour cell survival was assayed 18 h later. (○) Cyclophosphamide alone; (●) pyrazinamide + cyclophosphamide.

namely white blood cell counts, the ER was only 1.14 (± 0.08), which although not statistically significantly different from the ER for the RIF-1 tumour ($P > 0.05$) was different from the ER obtained in Lewis lung ($P < 0.02$). This suggests the potential for a therapeutic gain.

Cell killing by a variety of chemotherapeutic agents has been reported to be increased by structural analogues of pyrazinamide both *in vitro* (Durkacz *et al.*, 1980; Nduka *et al.*, 1980; Durrant & Boyle, 1982; Sakamoto *et al.*, 1983; Boorstein & Pardee, 1984; Brown *et al.*, 1984*b*; Jacobson *et al.*, 1984) and *in vivo* (Smulson *et al.*, 1977; Sakamoto *et al.*, 1983; Brown *et al.*, 1984*b*; Horsman *et al.*, 1986*b*). The enhancement of cytotoxicity *in vitro* has often been attributed to an inhibition of drug-induced potentially lethal damage (PLD) repair, but this may not explain the effects seen *in vivo*. When RIF-1 tumours were allowed to remain *in situ* after drug injection, prior to estimation of tumour cell killing, it was clearly demonstrated that this tumour possessed the ability to repair some of the damage induced by cyclophosphamide (Figure 3). While pyrazinamide enhanced the cell killing by this drug, it did so without inhibiting this PLD repair. Similar results were previously reported by us in the same tumour model using the alkylating agent melphalan and the structural analogue of pyrazinamide, nicotinamide (Horsman *et al.*, 1986*b*). The enhancement of melphalan cytotoxicity by nicotinamide, as well as by another analogue, 3-aminobenzamide, was almost entirely explained by a decrease in the rate of clearance of melphalan from mouse

**Table I** Characteristics of the dose–response curves for cyclophosphamide alone or cyclophosphamide and pyrazinamide[a]

| *Treatment*[b] | *Intercept*[c] | *Slope value* | *Slope ratio*[d] | *P value*[e] |
|---|---|---|---|---|
| *Lewis lung tumour* | | | | |
| CYT alone | | − 0.039 (± 0.003)[f] | | |
| | 1.47 (± 1.31) | | 1.54 (± 0.15) | $<1\times10^{-8}$ |
| PYZ + CYT | | − 0.060 (± 0.003) | | |
| *RIF-1 tumour* | | | | |
| CYT alone | | − 0.082 (± 0.004) | | |
| | 1.23 (± 1.26) | | 1.24 (± 0.08) | $<1\times10^{-8}$ |
| PYZ + CYT | | − 0.101 (± 0.004) | | |
| *WBC counts* | | | | |
| CYT alone | | − 0.012 (± 0.001) | | |
| | 5.80 (± 1.06) | | 1.14 (± 0.08) | <0.001 |
| PYZ + CYT | | − 0.013 (± 0.001) | | |

[a]Results are taken from the data shown in Figures 2 and 6. [b]The treatments were cyclophosphamide (CYT) ± pyrazinamide (PYZ), with the PYZ being injected 30 min (Lewis lung) or 120 min (RIF-1 and WBC counts) prior to CYT. [c]Each pair of survival curves had the same intercept. [d]This is the ratio of the slopes for CYT alone and CYT + PYZ in each tissue. [e]Represents the significance level of the difference between the two sets of slope values. [f]All values in parenthesis are standard errors.

**Figure 3** Survival of RIF-1 tumour cells as a function of time between cyclophosphamide administration and tumour removal. (△) Pyrazinamide (500 mg kg$^{-1}$); (○) cyclophosphamide (50 mg kg$^{-1}$); (●) pyrazinamide 2 h before cyclophosphamide.

**Figure 4** Mouse body temperature as a function of time after drug administration. Mice were injected with saline (S) or pyrazinamide (P; 500 mg kg$^{-1}$) 2 h prior to water (W) or cyclophosphamide (C; 75 mg kg$^{-1}$). Results show means (± 1 s.e.) for three mice from two separate experiments (different symbols). The dashed lines represent the range of values measured in control mice over the same time period.

**Figure 5** The effect of pyrazinamide (500 mg $kg^{-1}$) on the bioactivation of cyclophosphamide and the clearance of the cytotoxic species from mouse plasma. Lewis lung or RIF-1 tumour cells in culture were exposed for 3 h to plasma taken from mice at various times after injection with cyclophosphamide (75 mg $kg^{-1}$). Tumour cell survival was assayed immediately after drug exposure. (○) Cyclophosphamide alone; (●) pyrazinamide + cyclophosphamide. Pyrazinamide was injected 30 min before the alkylating agent in C57 mice (Lewis lung) or 2 h prior in C3H mice (RIF-1).

**Figure 6** Effect of pyrazinamide (500 mg $kg^{-1}$) on the number of white blood cell (WBC) counts in C3H mice measured 4 days after injection with different doses of cyclophosphamide. (○) Cyclophosphamide alone; (●) pyrazinamide 2 h before cyclophosphamide. Results show means (± 1 s.e.) for 5–6 mice per group.

plasma, an effect which may have partially been a consequence of hypothermia induced by both nicotinamide and 3-aminobenzamide. Pyrazinamide, on the other hand, had no effect on mouse or tumour temperature (Figure 4). Furthermore, it did not alter the bioactivation of cyclophosphamide, nor the clearance of this species from the plasma (Figure 5), thus some other mechanism must account for the chemosensitisation we observed.

No attempt was made by us to measure the pharmacokinetics or uptake of cyclophosphamide in the tumour, so any possible effect of pyrazinamide on these aspects cannot be ruled out. However, one possible explanation for the enhancement of the anti-tumour activity of cyclophosphamide by pyrazinamide may be improved drug delivery. Pyrazinamide and related compounds are known to be *in vivo* tumour radiosensitisers (Calcutt *et al.*, 1970; Jonsson *et al.*, 1985; Horsman *et al.*, 1986*a*, 1989*a*; Chaplin *et al.*, 1990*a*; Brown *et al.*, 1991; Kjellen *et al.*, 1991) and the mechanism for this effect has been suggested to be due to an improvement in tumour blood flow (Horsman *et al.*, 1988, 1989*b*; Chaplin *et al.*, 1990*a*; Lee & Song, 1992). More specifically, these agents actually improve oxygenation at the time of irradiation by preventing the transient stoppages in microregional blood flow (Chaplin *et al.*, 1990*a*, *b*; Horsman *et al.*, 1990) which have been shown to result in the development of acute hypoxia (Chaplin *et al.*, 1986). It is unlikely that the chemosensitisation produced by pyrazinamide in the current study is a consequence of simply increasing the oxygenation status of the tumour, since alkylating agents are generally considered to be equally toxic to both aerobic and hypoxic cells (Tannock & Guttman, 1981; Teicher *et al.*, 1981), although there is evidence that cells at reduced pH are more sensitive (Chaplin *et al.*, 1989), but in tumours these would typically be diffusion-limited chronically hypoxic cells and not those which are perfusion limited and thus acutely hypoxic. However, acutely hypoxic cells may actually be resistant to cyclophosphamide. The peak activity of cytotoxic species of cyclophosphamide in the plasma of both C3H and C57 mice was within 30 min following drug injection, and by 90–120 min no active drug could be identified (Figure 5). There is no definitive evidence showing exactly how long transient stoppages in blood flow can last in tumours, but it is possible that they could be of sufficient duration to actually prevent adequate drug exposure to some cells. Thus, as a result of temporary blood flow cessations, the acutely hypoxic cells may be protected from the cytotoxic action of cyclophosphamide. With pyrazinamide preventing these transient stoppages in flow the cyclophosphamide may then be able to reach cells in areas that perhaps normally it could not.

Whatever the explanation for the enhancement of cyclophosphamide cytotoxicity in tumours by pyrazinamide, this benzamide derivative is clearly an interesting compound and one that may have clinical applicability. In many respects it seems to have effects which are almost identical to the structural analogue nicotinamide, and this compound is now being considered for clinical testing as a radiation sensitiser. Pyrazinamide has already been used in humans, primarily as a secondary agent in the treatment of tuberculosis (Weinstein, 1975*b*). It is normally taken orally on a daily basis, in several equally spaced doses, for periods which can last for up to a few months. Side-effects limit the mean dose per day to around 3 g (Weinstein, 1975*a*). How 3 g in humans compares to the 500 $mg\ kg^{-1}$ mouse dose used in our current study is not known. Detailed pharmacokinetic analyses of nicotinamide in mice and humans have shown linear relationships between dose administered and peak plasma concentrations obtained, and that 3 g in humans resulted in the same peak plasma levels as a single dose of 75 $mg\ kg^{-1}$ in mice (Horsman *et al.*, 1993). Whether pyrazinamide has the same human to mouse relationship has not been determined. In fact there is evidence to suggest that the pharmacokinetics of pyrazinamide, at least in humans, differs dramatically from that of nicotinamide, since oral doses of 1, 2 or even 3 g of pyrazinamide have been reported to result in peak plasma concentrations that do not increase with dose; instead they appear to remain constant at around 40–50 $\mu g\ ml^{-1}$ (Stottmeier *et al.*, 1968; Weiner & Tinker, 1972). Clearly, this is an aspect that needs further investigation, as does additional *in vivo* testing of pyrazinamide as a radio- and chemosensitiser in other tumour models and appropriate normal tissues.

The authors wish to thank Mr D. Aoki, Mr D. Beagle, Mr W. Grulkey and Mr R. Miller for their skilled assistance with these experiments; Dr S.M. Bentzen for the statistical analysis; and Ms L. Spliid for preparation of the manuscript. This investigation was supported by PHS Grant No. CA-25990 from the National Cancer Institute of America, awarded to the Division of Radiation Biology at Stanford University School of Medicine, Stanford, CA 94305, USA (M.R.H.), and a grant from the National Cancer Institute of Canada, awarded to the Medical Biophysics Unit at the B.C. Cancer Research Center, Vancouver, Canada (D.J.C.).

### References

BEN-HUR, E., CHEN, C.C. & ELKIND, M. (1985). Inhibitors of poly(adenosine diphosphoribose) synthetase, examination of metabolic perturbations, and enhancement of radiation response in Chinese hamster cells. *Cancer Res.*, **45**, 2123–2127.

BOORSTEIN, R.J. & PARDEE, A.B. (1984). 3-aminobenzamide is lethal to MMS-damaged human fibroblasts primarily during S-phase. *J. Cell. Phys.*, **120**, 345–353.

BROWN, D.M., EVANS, J.W. & BROWN, J.M. (1984*a*). The influence of inhibitors of poly(ADP-ribose) polymerase on X-ray-induced potentially lethal damage repair. *Br. J. Cancer*, **49** (Suppl. VI), 27–31.

BROWN, D.M., HORSMAN, M.R., HIRST, D.G. & BROWN, J.M. (1984*b*). Enhancement of melphalan cytotoxicity *in vivo* and *in vitro* by inhibitors of poly (ADP-ribose) polymerase. *Int. J. Radiat. Oncol. Biol. Phys.*, **10**, 1665–1668.

BROWN, J.M., LEMMON, M.J., HORSMAN, M.R. & LEE, W.W. (1991). Structure activity relationships for tumor radiosensitization by analogs of nicotinamide and benzamide. *Int. J. Radiat. Biol.*, **59**, 739–748.

CALCUTT, G., TING, S.M. & PREECE, A.W. (1970). Tissue NAD levels and the response to irradiation on cytotoxic drugs. *Br. J. Cancer*, **24**, 380–388.

CHAPLIN, D.J., SHELDON, P.W., STRATFORD, I.J., AHMED, I. & ADAMS, G.E. (1983). Radiosensitization *in vivo*: a study with an homologous series of 2-nitroimidazoles. *Int. J. Radiat. Biol.*, **4**, 387–398.

CHAPLIN, D.J., DURAND, R.E. & OLIVE, P.L. (1986). Acute hypoxia in tumours: implication for modifiers of radiation effects. *Int. J. Radiat. Oncol. Biol. Phys.*, **12**, 1279–1282.

CHAPLIN, D.J., ACKER, B. & OLIVE, P.L. (1989). The potentiation of the tumor cytotoxicity of melphalan by vasodilating drugs. *Int. J. Radiat. Oncol. Biol. Phys.*, **16**, 1131–1135.

CHAPLIN, D.J., HORSMAN, M.R. & TROTTER, M.J. (1990*b*). Effect of nicotinamide on the microregional heterogeneity of oxygen delivery within a murine tumour. *J. Natl Cancer Inst.*, **82**, 672–676.

CHAPLIN, D.J., TROTTER, M.J., SKOV, K.A. & HORSMAN, M.R. (1990*a*). Modification of tumour radiation response *in vivo* by the benzamide analog pyrazinamide. *Br. J. Cancer*, **62**, 561–566.

COURTENAY, V.D. (1976). A soft agar colony assay for Lewis lung tumour and B16 melanoma taken directly from the mouse. *Br. J. Cancer*, **34**, 39–45.

DURKACZ, B.W., ONIDIJI, O., GRAY, D.A. & SHALL, S. (1980). (ADP-ribose)n participates in DNA excision repair. *Nature*, **283**, 593–596.

DURRANT, L.G. & BOYLE, J.M. (1982). Potentiation of cell killing by inhibitors of poly(ADP-ribose)polymerase in four rodent cell lines exposed to N-methyl-N-nitrosourea or UV light. *Chem. Biol. Interact.*, **38**, 325–338.

HORSMAN, M.R., EVANS, J.W. & BROWN, J.M. (1984). Enhancement of melphalan-induced tumour cell killing by misonidazole: an interaction of competing mechanisms. *Br. J. Cancer*, **50**, 305–316.

HORSMAN, M.R., BROWN, D.M., LEMMON, M.J., BROWN, J.M. & LEE, W.W. (1986*a*). Preferential tumour radiosensitization by analogs of nicotinamide and benzamide. *Int. J. Radiat. Oncol. Biol. Phys.*, **12**, 1307–1310.

HORSMAN, M.R., BROWN, D.M., HIRST, D.G. & BROWN, J.M. (1986*b*). Changes in the response of the RIF-1 tumour to melphalan *in vivo* induced by inhibitors of nuclear ADP-ribosyl transferase. *Br. J. Cancer*, **53**, 247–254.

HORSMAN, M.R., BROWN, J.M., HIRST, V.K., LEMMON, M.J., WOOD, P.J., DUNPHY, E.P. & OVERGAARD, J. (1988). Mechanism of action of the selective tumor radiosensitizer nicotinamide. *Int. J. Radiat. Oncol. Biol. Phys.*, **15**, 685–690.

HORSMAN, M.R., HANSEN, P.V. & OVERGAARD, J. (1989*a*). Radiosensitization by nicotinamide in tumors and normal tissues: the importance of tissue oxygenation status. *Int. J. Radiat. Oncol. Biol. Phys.*, **16**, 1273–1276.

HORSMAN, M.R., CHAPLIN, D.J. & BROWN, J.M. (1989*b*). Tumor radiosensitization by nicotinamide: a result of improved blood perfusion and oxygenation. *Radiat. Res.*, **118**, 139–150.

HORSMAN, M.R., CHAPLIN, D.J. & OVERGAARD, J. (1990). Combination of nicotinamide and hyperthermia to eliminate radioresistant chronically and acutely hypoxic tumor cells. *Cancer Res.*, **50**, 7430–7436.

HORSMAN, M.R., KRISTJANSEN, P.E.G., MIZUNO, M., CHRISTENSEN, K., CHAPLIN, D.J., QUISTORFF, B. & OVERGAARD, J. (1992). Biochemical and physiological changes induced by nicotinamide in a C3H mouse mammary carcinoma and CDF1 mice. *Int. J. Radiat. Oncol. Biol. Phys.*, **22**, 451–454.

HORSMAN, M.R., HØYER, M., HONESS, D.J., DENNIS, I.F. & OVERGAARD, J. (1993). Nicotinamide pharmacokinetics in humans and mice: a comparative assessment and the implications for radiotherapy. *Radiother. Oncol.*, **27**, 131–139.

JACOBSON, E.L., SMITH, J.Y., MINGMUANG, M., MEADOWS, R., SIMS, J.L. & JACOBSON, M.K. (1984). Effect of nicotinamide analogues on recovery from DNA damage in C3H 10T 1/2 cells. *Cancer Res.*, **44**, 2485–2492.

JONSSON, G.G., KJELLEN, E., PERO, R.W. & CAMERON, R. (1985). Radiosensitization effects of nicotinamide on malignant and normal mouse tissues. *Cancer Res.*, **45**, 3609–3614.

KJELLEN, E., JOINER, M.C., COLLIER, J.M., JOHNS, H. & ROJAS, A. (1991). A therapeutic benefit from combining normobaric carbogen or oxygen with nicotinamide in fractionated X-ray treatments. *Radiother. Oncol.*, **22**, 81–91.

LAW, M.P., HIRST, D.G. & BROWN, J.M. (1981). The enhancing effect of misonidazole on the response of the RIF-1 tumour to cyclophosphamide. *Br. J. Cancer*, **44**, 208–218.

LEE, I. & SONG, C.W. (1992). The oxygenation of murine tumor isografts and human tumor xenografts by nicotinamide. *Radiat. Res.*, **130**, 65–71.

NDUKA, N., SKIDMORE, C.J. & SHALL, S. (1980). The enhancement of cytotoxicity of N-methyl-N-nitrosourea and of γ-radiation by inhibitors of poly(ADP-ribose) polymerase. *Eur. J. Biochem.*, **105**, 525–530.

SAKAMOTO, H., KAWAMITSU, H., MIWA, M., TERADA, M. & SUGIMURA, T. (1983). Enhancement of antitumour activity of bleomycin by benzamide *in vitro* and *in vivo*. *J. Antibiotics*, **36**, 296–301.

SMULSON, M.E., SCHEIN, P., MULLINS, D.W. & SUDHAKAR, S. (1977). A putative role for nicotinamide adenine dinucleotide-promoted nuclear protein modification in the antitumour activity of N-methyl-N-nitrosourea. *Cancer Res.*, **37**, 3006–3012.

STOTTMEIER, K.D., BEAM, R.E. & KUBICA, G.P. (1968). The absorption and excretion of pyrazinamide. I. Preliminary study in laboratory animals and in man. *Am. Rev. Respir. Dis.*, **98**, 70–74.

TANNOCK, I.F. & GUTTMAN, P. (1981). Response of Chinese hamster ovary cells to anticancer drugs under aerobic and hypoxic conditions. *Br. J. Cancer*, **43**, 245–248.

TEICHER, B.A., LAZO, J.S. & SARTORELLI, A.C. (1981). Classification of antineoplastic agents by their selective toxicities toward oxygenated and hypoxic tumor cells. *Cancer Res.*, **41**, 73–81.

TWENTYMAN, P.R., BROWN, J.M., GRAY, J.W., FRANKO, A.J., SCOLES, M.A. & KALLMAN, R.F. (1980). A new mouse tumour model system (RIF-1) for comparison of end-point studies. *J. Natl Cancer Inst.*, **64**, 595–604.

WEINER, I.M. & TINKER, J.P. (1972). Pharmacology of pyrazinamide: Metabolic and renal function studies related to the mechanism of drug-induced urate retention. *J. Pharm. Exp. Ther.*, **180**, 411–434.

WEINSTEIN, L. (1975*a*). Antimicrobial agents: drugs used in the chemotherapy of tuberculosis and leprosy. In *The Pharmacological Basis of Therapeutics*. 5th edn., Goodman, L.S. & Gilman, A. (eds), pp. 1201–1223. Macmillan: New York.

WEINSTEIN, L. (1975*b*). Antimicrobial agents: general considerations. In *The Pharmacological Basis of Therapeutics*, 5th edn., Goodman, L.S. & Gilman, A. (eds), pp. 1090–1112. Macmillan: New York.